# Effect of Ivermectin Treatment on the Frequency of Seizures in Persons with Epilepsy Infected with *Onchocerca volvulus*

**DOI:** 10.3390/pathogens10010021

**Published:** 2020-12-31

**Authors:** Alfred Dusabimana, Solomon Tsebeni Wafula, Stephen Jada Raimon, Joseph Nelson Siewe Fodjo, Dan Bhwana, Floribert Tepage, Gasim Abd-Elfarag, An Hotterbeekx, Steven Abrams, Robert Colebunders

**Affiliations:** 1 Global Health Institute, University of Antwerp, Doornstraat 331, 2610 Antwerp, Belgium; swafula@musph.ac.ug (S.T.W.); josephnelson.siewefodjo@uantwerpen.be (J.N.S.F.); an.hotterbeekx@uantwerpen.be (A.H.); steven.abrams@uantwerpen.be (S.A.); 2 Department of Disease Control and Environmental Health, Makerere University, Kampala P.O. Box 7072, Uganda; 3 Amref Health Africa, Juba P.O. Box 30125, South Sudan; Stephen.Jada@amref.org; 4 National Institute of Medical Research, Tanga Centre, Tanga P.O. Box 5004, Tanzania; dan.bhwana@nimr.or.tz; 5 Ministry of Health, Bas Uélé Province, Buta B.P. 105, Democratic Republic of Congo; floritepage@yahoo.fr; 6 Amsterdam Institute for Global Health and Development, Paasheuvelweg 25, 1105 BP Amsterdam, The Netherlands; gasim4u83@gmail.com; 7 Data Science Institute, Interuniversity Institute for Biostatistics and Statistical Bioinformatics, Hasselt University, 3590 Diepenbeek, Belgium

**Keywords:** epilepsy, ivermectin, onchocerciasis, seizures

## Abstract

A clinical trial performed in the Democratic Republic of Congo (DRC), among persons with epilepsy (PWE) infected with *Onchocerca volvulus* treated with anti-seizure medication suggested that ivermectin reduces the seizure frequency. We assessed the effect of ivermectin treatment on seizure frequency in PWE with and without anti-seizure medication in three onchocerciasis endemic areas (Maridi, South Sudan; Aketi, DRC; and Mahenge, Tanzania). Pre- and 3–5 months post-ivermectin microfilariae densities in skin snips and seizure frequency were assessed. After ivermectin, the median (IQR) percentage reduction in seizure frequency in the study sites ranged from 73.4% (26.0–90.0) to 100% (50.0–100.0). A negative binomial mixed model showed that ivermectin significantly reduced the seizure frequency, with a larger decrease in PWE with a high baseline seizure frequency. Mediation analysis showed that ivermectin reduced the seizure frequencies indirectly through reduction in microfilariae densities but also that ivermectin may have a direct anti-seizure effect. However, given the short half-life of ivermectin and the fact that ivermectin does not penetrate the healthy brain, such a direct anti-seizure effect is unlikely. A randomized controlled trial assessing the ivermectin effect in people infected with *O. volvulus* who are also PWE on a stable anti-seizure regimen may be needed to clarify the causal relationship between ivermectin and seizure frequency.

## 1. Introduction

The filarial nematode *Onchocerca volvulus* (*O. volvulus*) is known to cause skin disease, and ocular problems (river blindness). There is growing evidence that this parasite directly or indirectly can cause nodding syndrome as well as other forms of epilepsy (onchocerciasis-associated epilepsy or OAE) [1].

Ivermectin is currently the drug of choice to treat human onchocerciasis [2]. Although ivermectin does not kill the adult filarial worm, it decreases its fertility, kills the microfilariae, and has an excellent safety profile with adverse events occurring only as a consequence of an immunological response against the killed microfilariae [3]. These effects termed as “Mazzotti reactions” are usually mild and disappear within days without further treatment [4,5]. 

Annual or bi-annual community-directed treatment with ivermectin (CDTI) is the cornerstone of onchocerciasis elimination programs [6]. One dose of ivermectin of 150 to 200 µg/kg body weight eliminates microfilariae very rapidly [7]. A mathematical model predicted that microfilariadermia would be reduced by half after 24 h, by 85% after 72 h, and by 94% one week after the intake of ivermectin [7]. If microfilarial density plays a role in causing OAE, it is expected that a reduction in microfilarial density following treatment with ivermectin may also be able to decrease the frequency of seizures.

Until recently there was only anecdotal evidence suggesting that ivermectin use in onchocerciasis-endemic regions reduces the frequency of seizures. In 1992, a study in Kabarole reported a decrease in either frequency or severity of seizures after a single dose of ivermectin at 150 µg/kg in 34 out of 91 persons with epilepsy (PWE) [8]. After being treated with ivermectin, 13 (14%) individuals experienced no seizures for 3.7 months on average; seizures were unchanged in 51 (56%) and worsened in 6 (7%) [8]. A positive correlation between seizure frequency and microfilarial density was reported previously in the Aketi health zone, Bas Uélé province and the Logo health zone in Ituri province, onchocerciasis endemic regions in the Democratic Republic of Congo (DRC) [9]. A proof of concept randomised trial to investigate whether ivermectin had an added value in reducing the frequency of seizures in persons with onchocerciasis-associated epilepsy (OAE) also treated with anti-epileptic drugs, showed a borderline association between ivermectin treatment and seizure-freedom at 4 months of the trial [10]. Upon prolonging the follow-up period of this initial trial to one year while providing additional doses of ivermectin to some participants, seizure freedom during the last four months of follow-up was more likely among those treated with ivermectin twice (OR: 5.09, 95% CI: 1.38–19.75) and thrice (OR: 2.47, 95% CI: 0.94–6.77) than in those treated once [11]. 

Recently, we investigated the ivermectin response on *O. volvulus* microfilarial densities in persons with epilepsy (PWE) in four onchocerciasis-endemic areas in Africa, with and without a history of CDTI. A higher pre-ivermectin microfilarial density was associated with increased probability of a positive skin snip after ivermectin treatment and among the participants with persistent microfiladermia during follow-up, a higher number of previous community-based treatment rounds with ivermectin increased the probability of having a post-treatment microfilarial density >20% of the pre-treatment value [12]. In the present paper, we study the effect of ivermectin on the frequency of seizures in people infected with *O. volvulus* who are also PWE whereby some individuals were on anti-epileptic treatment and others were not. 

## 2. Materials and Methods 

### 2.1. Study Setting and Participants

We carried out three pre–post-observational studies of PWE with an *O. volvulus* infection treated with ivermectin in three different sites: one in the DRC, one in Tanzania and another in South Sudan. These are all rural settings with a high prevalence of epilepsy and onchocerciasis. 

#### 2.1.1. Aketi Health Zone, DRC 

In 2015, a high prevalence of epilepsy was documented in Wela (6.5%) and Makoko (7.8%), two villages in the Aketi health zone, an onchocerciasis hyper-endemic area in the Bas-Uélé province in the DRC [13]. CDTI had been initiated in this health zone for 14 years. In 2017, an epilepsy prevalence of 5.7% was also observed in Aketi rural town together with a 64.5% seroprevalence of OV16 antibodies in children aged 7–10 years old, suggesting high ongoing onchocerciasis transmission [14]. PWE were selected in the villages of Wela, Makoko and Aketi rural town. All individuals received a single dose of ivermectin and skin snip testing was performed before ivermectin treatment and repeated after three months. 

#### 2.1.2. Maridi, South Sudan 

In May 2018, during a door-to-door household survey in 8 villages in an onchocerciasis endemic area in Maridi County, an overall epilepsy prevalence of 4.4% was documented with the highest epilepsy prevalence of 11.9% in a village close to the Maridi dam, a blackfly breeding site [15]. CDTI had been interrupted for many years and was re-initiated in 2017. In December 2018, PWE identified during the survey in May were asked to participate in this study. After an initial skin snip, the participants received a single dose of ivermectin and skin snip testing was repeated after five months.

#### 2.1.3. Mahenge, Ulanga District, Tanzania 

The National Onchocerciasis Control Programme has conducted CDTI in Mahenge since 1997. In January 2017, an epilepsy prevalence survey was conducted in two rural villages in the Mahenge area (Mdindo, Msogezi), and documented a high prevalence of epilepsy of 3.5% together with evidence of ongoing *O. volvulus* transmission (42.6% children 6–10 years old presented with OV16 antibodies) [16]. PWE identified during this survey were asked to participate in the study. A first skin snip was obtained, after which all participants received a single dose of ivermectin, and skin snip testing was repeated after three months.

### 2.2. Skin Snip Testing

Skin snips were obtained from each posterior iliac crest of eligible participants using a Holt-type punch. Snips were immediately placed in two wells of a microtitre plate containing 3 drops of normal saline solution and incubated for 24 h at room temperature to allow microfilariae to emerge into the fluid. After the incubation period, mf in the solution were examined microscopically under a ×40 magnification and counted by a trained technician. Microfilarial (mf) densities of positive samples were expressed as the arithmetic mean from both right and left skin snips (mean microfilariae/skin snip). One punch was used per subject and punches were sterilized between subjects using steam under pressure (autoclave). Only PWE with positive skin snips at baseline were included in this study. 

### 2.3. Ivermectin Treatment 

All ivermectin tablets (3 mg) were taken orally once under direct observation according to participant height, as recommended by the World Health Organization [17,18].

### 2.4. Medical History and Clinical Examination

PWE and their caretakers were questioned about seizure frequency per day, per week, and per month during the month preceding ivermectin intake. Information was also obtained regarding the use of anti-seizure drugs, and past ivermectin intake. Three to five months after ivermectin treatment, the seizure frequency assessment was repeated. 

### 2.5. Statistical Analysis 

As the primary objective was to evaluate the effect of ivermectin treatment on seizure frequency, assessed at two time points (before ivermectin intake and 3–5 months after ivermectin treatment depending on the study site under consideration), this analysis included PWE with at least one seizure in the month preceding ivermectin intake. Continuous variables were summarized using the median and interquartile range (IQR). Categorical data were reported using absolute and relative proportions. The percentage reduction in frequency of seizures was calculated as the difference between pre- and post-ivermectin values divided by the pre-ivermectin values multiplied by 100. Pre- and post-ivermectin seizure frequencies were compared using the Wilcoxon signed-rank test. We assumed that the seizure frequency conditional on covariates follows a negative binomial distribution. This distribution is an extension of the Poisson distribution commonly used to model count data. We used a negative binomial mixed model to obtain marginal estimates of the effect of ivermectin on the frequency of seizures while adjusting for relevant individual- and community-level covariates. The negative binomial mixed model included individual-specific random effects to accommodate association in the data as a result of repeated measurements at the same individual. The covariates included gender, age, study site, anti-seizure drug use, previous ivermectin use, and baseline mf density per skin snip. Ivermectin treatment was represented by time period (pre- and post-ivermectin). All possible two-way interactions between covariates were checked to be included in the final model, and inclusion into the model was determined based on the likelihood ratio test.

We conducted a mediation analysis to determine the effect of ivermectin itself (direct effect) and the effect of ivermectin through the reduction in mf density (indirect effect) on seizure frequency. Assuming that all confounders are controlled in the model and there are no time-varying confounders with respect to ivermectin and microfilarial density, we used the mediation analysis method for longitudinal data proposed by Bind et al., [19] available in the statistical software program SAS. We estimated the asymptotic sampling variances of the direct and indirect effects of ivermectin on seizure frequency using bootstrapping procedures. To preserve the correlation between pre- and post-ivermectin seizures and mf density, we resampled the longitudinal data with replacement. Unstandardized direct and indirect effects were computed for each of 500 bootstrapped samples, and bootstrap-based 95% percentile confidence intervals were computed. Data were analysed using R version 4.0.2, and SAS 9.4 (SAS Institute Inc., Cary, NC, USA).

### 2.6. Ethical Approval and Consent to Participate 

Ethical approvals were obtained from the ethics committees of the University of Antwerp (Belgium), the ethical committee of the School of Public Health, Kinshasa (DRC), the Ministry of Health of the Republic of South Sudan, and the National Institute of Medical Research, Tanzania. The purpose and procedures of the study were explained to all participants in their local languages. Participants were free to abstain from participation in the study or to withdraw consent to participate at any time. No direct benefits for participation in the study were provided. All participants were asked to sign an informed consent form and only consenting individuals were enrolled. Minors >12 years and <18 years signed an assent form, while parents or legal guardians consented for younger participants. All individual data were encoded and treated confidentially. 

## 3. Results

### 3.1. Baseline Characteristics of Participants

A total of 215 PWE from three study sites participated: Maridi, South Sudan (*n*  =  105; 48.8%), Aketi, DRC (*n*  =  87; 40.5%), and Mahenge, Tanzania (*n*  =  23; 10.7%). About half of the study participants (48.8%) were males. Of these, 35 (16%) PWE had less than one seizure per month during the month preceding ivermectin intake. Generalized tonic-clonic seizures were the most frequent type of seizures in the three study sites. The median age was 17.0 years (IQR: 15.0–21.0) (Table 1).

### 3.2. Pre- and Post-Ivermectin Microfilarial Density and Seizure Frequency

In the Aketi health zone, three months after ivermectin intake, 77.8% (63/81) of PWE were cleared of microfilariae and had negative skin snips. The median (IQR) % reduction in seizure frequency three months after ivermectin intake was 100% (50.0–100.0) (Table 2).

In Maridi, five months after ivermectin intake, 21.0% (22/105) of PWE were free from microfilariae in skin snips. Median seizure frequency reduced significantly between baseline and follow up. (Table 3).

In Mahenge, three months after ivermectin intake, 52.2% (12/23) of PWE were free from microfilariae in skin snips. Median mf density reduced significantly after ivermectin intake. The median % reduction in seizure frequency was 100% (85.8–100.0) (Table 4). 

Combining the results of the three study sites, the median (IQR) seizure frequency per month was 6.0 (2.0–60.0) before ivermectin treatment and 3.0 (1.0–16.0) after ivermectin treatment. There was a significant difference between median frequency of seizure before and after ivermectin treatment (sign test statistic = 62, *p*-value ≤ 0.001). 

**Figure 1:**
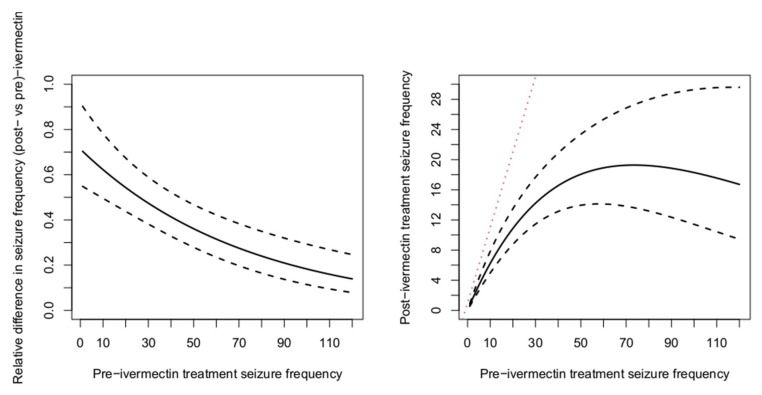
The relative difference in post- vs pre-ivermectin seizure frequency as a function of pre-ivermectin seizure frequency. The solid black line represents the estimated relative difference and dashed lines indicate pointwise 95% confidence bands computed based on estimated variance-covariance matrix of the parameter (left panel). The right panel shows the expected seizure frequency rate after ivermectin treatment as a function of the baseline seizure frequency (solid black line); 95% confidence limits are displayed using dashed lines. Red dotted line displays no effect of ivermectin treatment (i.e., frequency of seizure before ivermectin treatment = frequency of seizure after ivermectin treatment).

According to the negative binomial mixed effect model, the expected seizure frequency significantly decreased after ivermectin treatment with a larger decrease in PWE with higher baseline seizure frequencies (Table 5 and Figure 1). In the left panel of Figure 1, we present the relative difference in seizure frequency as a function of the baseline seizure frequency (i.e., the ratio of seizure frequency after vs. before ivermectin treatment). The relative difference decreases monotonically with increasing baseline seizure frequency. The number of seizures after ivermectin treatment as a function of the baseline seizure frequency is depicted in the right panel of Figure 1. More specifically, for persons with ten seizures at baseline, 60% of the seizures, or six seizures, are on average observed after ivermectin treatment.

After adjusting for age, gender, history of ivermectin, past exposure to anti-seizure drug and study site, both the direct and indirect ivermectin effect on the frequency of seizures was significant (Table 6). A schematic representation of the decomposition of the ivermectin effect in a direct and indirect effect is presented in Figure S1 of the Supplementary Material.

## 4. Discussion

This study assessed the effect of ivermectin on the frequency of seizures among PWE living in onchocerciasis-endemic areas. Our findings demonstrate that ivermectin treatment intake reduces the frequency of seizures in people infected with *O. volvulus* who are also PWE, three to five months after treatment. PWE with a higher pre-ivermectin seizure frequency experienced a larger reduction in post-ivermectin seizure frequency. 

PWE in Maridi presented higher post-ivermectin seizure frequency as compared to those from Aketi. This could be due to the higher pre-ivermectin mf density among PWE in Maridi, which was probably due to the prolonged CDTI interruption in South Sudan. High mf densities have been shown to be associated with more severe forms of OAE, with more seizures and disabilities [20]. In a recent randomised trial among people infected with *O. volvulus* who are also PWE, seizure freedom was more often observed in the last 4 months of a one year trial in those who received two or three doses of ivermectin compared to only one dose [11]. This difference was explained by a higher mf load during this period in the one dose treatment arm. The probability of being seizure-free was found to be positively associated with the absence of mf; OR = 2.618 (95% CI: 1.136–6.289). A problem with this trial was that at enrolment all PWE were not on a regular anti-seizure treatment and that during the trial, anti-seizure drug doses frequently had to be adapted, complicating the interpretation of the trial results.

In our three-country study, because of the lack of a control group and the problem with potential confounders, it was difficult to investigate the relationship between the effect of ivermectin on mf load and frequency of seizures. However, using mediation analysis, both the direct and indirect effects of ivermectin led to a significant reduction in post-ivermectin treatment seizure frequency. This suggests that next to the reduction in mf density and the effect thereof on the seizure frequency, ivermectin treatment itself may have an additional beneficial effect in lowering the frequency of seizures. An anti-convulsive effect of ivermectin is unlikely because, at the therapeutic dose, ivermectin is unable to cross the human blood–brain barrier [21]. Moreover, given the short half-life of ivermectin (about 56.50 ± 7.01 h) [22], it is difficult to explain how one dose of ivermectin would have a direct anti-seizure effect during 3–5 months. An anti-convulsive effect of ivermectin was suggested in a small non-randomised trial conducted in Spain among individuals with refractory epilepsy [23]. However, in the latter study, ivermectin was given three or seven times a week at a 10 mg/day dose along with anti-seizure drugs, and some patients had brain lesions which possibly compromised the integrity of the blood–brain barrier. It is unclear how ivermectin may have had a direct anti-seizure effect in our study. In the informed consent of the study, we mentioned that one of the study objectives was to determine whether ivermectin is able to decrease the frequency of seizures. Therefore, it is possible that the observed decrease in seizures was partially caused by a placebo effect. On the other hand, the relation between seizure frequency and mf density, prior to and after ivermectin treatment, is not fully captured by the model, thereby inducing a direct effect of ivermectin to explain the entire extent of the reduction in seizure frequency. Quantification of seizure frequency and mf density based on a single measurement before and after ivermectin treatment potentially introduces additional variability, masking the relation between frequency of seizures and mf density, at least partly. 

A strength of our study is that similar results were obtained in three different study sites by three different teams of investigators. However, our results have to be interpreted in light of some limitations. The information on seizure frequency and anti-seizure drug use was obtained by interviewing PWE/family members, and therefore, we cannot rule out recall bias. We did not obtain detailed information about anti-seizure drug use (including treatment adherence) prior to the ivermectin intake and during the follow-up period. Mf densities were only measured per skin snip and not per mg of skin, rendering standardization and comparison across sites difficult. Moreover, our analysis included only *O. volvulus* infected PWE who came back for a second skin snip test. The lack of information on frequency of seizures of PWE who did not came back for a follow up could have influenced the study results. Indeed, some of them may not have come back because they did not observe any improvement in frequency of seizures thereby potentially biasing the estimated ivermectin effect on seizure frequency. Finally, no additional laboratory studies, nor neuro-imaging studies were performed to determine the aetiology of the epilepsy.

In conclusion, PWE with *O. volvulus* infection were found to have fewer seizures 3–5 months after receiving ivermectin, suggesting that ivermectin is effective in reducing seizures. Our finding that ivermectin is able to reduce the frequency of seizures through the reduction in mf density confirms the results of a recently performed clinical trial that investigated the effect of ivermectin on the frequency of seizures. However, our study suggests that the decrease in seizure frequency following ivermectin treatment cannot solely be ascribed to a reduction in mf density. Given the short half-life of ivermectin and the fact that ivermectin does not penetrate the brain, a direct anti-seizure effect of ivermectin is unlikely. A randomized controlled trial evaluating the effect of ivermectin in people infected with *O. volvulus* who are also PWE on a stable anti-seizure regimen may be needed to clarify the causal relationship between ivermectin use and the frequency of seizures.

## Figures and Tables

**Table 1:** Baseline characteristics of people infected with onchocerciasis who are also persons with epilepsy (PWE).

Characteristic	Maridi (*n* = 105)	Aketi (*n* = 87)	Mahenge (*n* = 23)	All Participants (*n* = 215)
Age (years): median (IQR)	17.0 (14.0–18.0)	17.0 (15.0–20.0)	33.0 (27.3–42.0)	17.0 (15.0–21.0)
Gender: Male, *n* (%)	53 (50.5)	40 (49.4)	9 (39.1)	102 (48.8)
Weight (kg): median (IQR)	37.0 (30.0–48.0)	39.0 (30.0–43.0)	45.0 (40.5–49. 5)	40.0 (31.0–48.0)
History of anti-seizure drug use at enrolment, *n* (%)	97 (93.3)	55 (63.2)	21 (95.5)	173 (81.2)
Most frequent types of seizure				
Generalized tonic-clonic seizures: *n* (%)	58 (55.2)	74 (85.1)	17 (73.9)	149 (69.3)
Atonic seizures (drop attacks): *n* (%)	1 (0.9)	0 (0.0)	1 (4.3)	2 (0.9)
Absences: *n* (%)	3 (2.9)	4 (4.6)	3 (13.0)	10 (4.6)
Nodding seizures: *n* (%)	25 (23.8)	7 (8.0)	2 (8.7)	34 (15.8)
Generalized tonic-clonic with nodding seizures: *n* (%)	18 (17.1)	2 (2.3)	0 (0.0)	20 (9.3)
Ivermectin use in 2018, *n* (%)	41 (39.0)	81 (93.1)	18 (78.3)	140 (65.1)

**Table 2:** Ivermectin response in 87 people infected with onchocerciasis who are also PWE in Aketi, DRC.

Variables	Before Ivermectin Intake	3 Months after Ivermectin Intake	*p*-Value
Positive skin snip for mf: *n* (%)	87 (100)	18 (22.2)	<0.001 ^a^
mf density: median (IQR)	12.0 (4.0–63.0)	0.0 (0.0–0.0)	<0.001 ^b^
Seizures per month: median (IQR)	1.0 (0.5–2.0)	1.0 (0.0–2.0)	<0.001 ^b^
Number of PWE with <1 seizure per month: *n* (%)	10 (11.5)	55 (63.3)	<0.001 ^a^
% reduction in frequency of seizures: median (IQR)	NA	100 (50.0–100)	NA

^a^*p*-values based on McNemar Test; ^b^*p*-values based on Wilcoxon signed-rank test; mf = microfilarial density.

**Table 3:** Ivermectin response in people infected with onchocerciasis who are also PWE in Maridi, South Sudan.

Variables	Before Ivermectin Intake	5 Months after Ivermectin Intake	*p*-Value
Positive skin snip for mf: *n* (%)	105 (100)	83/105 (79.0%)	<0.001 ^a^
mf density: median (IQR)	52.0 (29.0–84.0)	3.0 (1.0–10.0)	<0.001 ^b^
Seizures per month: median (IQR)	12.0 (2.0–90.0)	8.0 (2.0–20.0)	<0.001 ^b^
Number of PWE with <1 seizure per month: *n* (%)	8 (7.6)	45 (42.9)	NA
% reduction in frequency of the seizures: median (IQR)	NA	73.4 (26.0–90.0)	NA

^a^*p* values based on McNemar Test; ^b^ *p* values based on Wilcoxon signed ranked test; mf = microfilarial density.

**Table 4:** Ivermectin response in people infected with onchocerciasis who are also PWE in Mahenge, Tanzania.

Parameters	Before Ivermectin Intake	3 Months after Ivermectin Intake	*p*-Value
Positive skin snip for mf: *n* (%)	23 (100)	11/23 (47.8)	0.002 ^a^
mf density: median (IQR)	3.0 (1.0–5.0)	0.0 (0.0–1.0)	<0.001 ^b^
Seizures per month: median (IQR)	2.0 (1.0–2.0)	0.0 (0.0–1.0)	0.093 ^b^
Number of PWE with <1 seizure per month: *n* (%)	2 (8.7)	12 (52.2)	<0.001 ^a^
% reduction in frequency of seizures: median (IQR)	NA	100 (85.8–100.0)	NA

^a^*p*-values based on McNemar Test; ^b^*p*-values based on Wilcoxon signed-rank test; mf = microfilarial density.

**Table 5:** Predictors of seizure frequency using a negative binomial mixed model.

Effect	Estimate	95% CI		*p*-Value
Intercept	2.322	0.736	3.908	0.004
Age (years)	−0.048	−0.107	0.012	0.114
Gender (male vs female)	−0.309	−0.833	0.215	0.245
Site (Mahenge vs Aketi)	−0.610	−2.207	0.987	0.452
Site (Maridi vs Aketi)	1.646	0.908	2.385	<0.001
Baseline mf/skin snip	0.002	−0.003	0.006	0.471
After ivermectin treatment (vs before)	−0.338	−0.592	−0.084	0.009
Seizure before ivermectin * After ivermectin treatment (vs before)	−0.014	−0.019	−0.008	<0.001
Previous ivermectin (used vs not used)	−0.371	−1.009	0.268	0.253
Anti-seizure drug use with ivermectin	0.144	−0.436	0.724	0.624
Var[b0] (se)	2.129 (0.325)			

mf = microfilarial density; Var {b0}: Variance of random intercepts; se: standard error; *: interaction effect.

**Table 6:** Causal mediation effects of microfilarial density.

Estimates	Estimate	95% CI	
Indirect effect	−0.014	−0.020	−0.006
Direct effect	−0.715	−0.955	−0.465
Total effect	−0.730	−0.970	−0.470
Percentage mediated	1.867	1.363	2.087

CI: 95% bootstrap confidence interval.

## Data Availability

The datasets generated during the current study are available from the corresponding authors on reasonable request.

## Supplementary Materials

The following are available online at https://www.mdpi.com/2076-0817/10/1/21/s1, Figure S1: Mediation pathways.
