# Supplementary material for: Effect of Ivermectin Treatment on the Frequency of Seizures in Persons with Epilepsy Infected with Onchocerca volvulus
Source: Pathogens. 2020 Dec 31;10(1):21. doi: 10.3390/pathogens10010021 (PMC7824398; doi:10.3390/pathogens10010021)
Supplement: Supplementary file 1 [file pathogens-10-00021-s001.pdf]

Supplementary Material: Mediation pathways

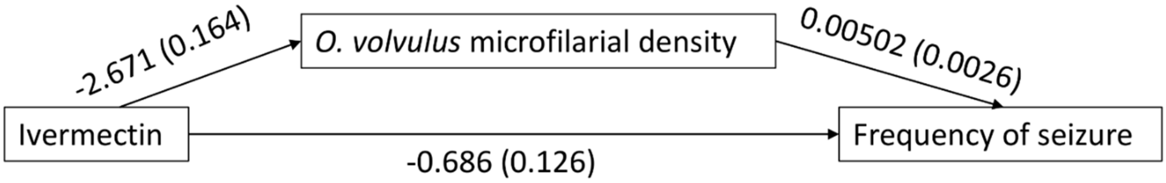

Figure S1. Mediation pathways.

The indirect effect was given by (-2.671)*(0.00502) = -0.0134. The bootstrapped unstandardized indirect effect was -0.0136, with 95% confidence interval from -0.0202 to -0.0064. Thus, the indirect effect was statistically significant (p-value <0.001). The ivermectin effect either mediated by participant-specific mf density (indirect effect) or unmediated (direct effect) leads to a significant reduction in frequency of seizures. In conclusion, both the effect of ivermectin itself (direct effect) and the effect of ivermectin through the reduction in mf density (indirect effect) on frequency of seizure were found to be significant (see also Table 6 in the main text).
